# Breast milk and infant formula milk for the prevention of hypoxic-ischemic encephalopathy in premature infants

**DOI:** 10.1192/j.eurpsy.2021.2012

**Published:** 2021-08-13

**Authors:** G. Abdullayeva, M. Abdullaeva

**Affiliations:** 1 Department Of Propaedeutic Of Childhood Diseases, Kazakh National Medical University after Asfendiyarov, Almaty, Kazakhstan; 2 Psychology, Lomonosov Moscow State University, Moscow, Russian Federation

**Keywords:** premature infants, breast milk, mental health, hypoxic-ischemic encephalopathy

## Abstract

**Introduction:**

A relationship was found between the use of breast milk and infant formula milk and a decrease in the incidence and number of clinical complications caused by hypoxic-ischemic encephalopathy hypoxic-ischemic encephalopathy.

**Objectives:**

To assess the efficacy and safety of breast milk and infant formula milk in terms of reducing of hypoxic-ischemic encephalopathy, the level of morbidity, the severity of damage to brain structures, the time before switching to full-fledged enteral nutrition and the frequency of detecting feeding intolerance in premature infants.

**Methods:**

Prospective observation of the development of 254 premature babies were treated up to six months of corrected age at the Department of Neurology of Early Growth in 2016-2018. The effect of breast milk and formula milk on neurological status was compared.

**Results:**

In comparison, breast milk and formula milk didn’t show any effect on the frequency of severe hypoxic-ischemic encephalopathy (p <0,05), the severity of brain damage(р <0,01). Breast milk showed a statistically significant effect in terms of reducing the average number before switching to full enteral nutrition (p <0,01).

**Conclusions:**

Breast milk and formula milk does not affect the frequency of development of hypoxic-ischemic encephalopathy, the severity of brain damage. Breast milk significantly reduces the frequency of feeding intolerance, accelerates the transition to enteral nutrition, reduces the duration of hospitalization in premature infants.

**Disclosure:**

No significant relationships.

